# Feedback modulation of neural network synchrony and seizure susceptibility by Mdm2-p53-Nedd4-2 signaling

**DOI:** 10.1186/s13041-016-0214-6

**Published:** 2016-03-22

**Authors:** Kathryn A. Jewett, Catherine A. Christian, Jonathan T. Bacos, Kwan Young Lee, Jiuhe Zhu, Nien-Pei Tsai

**Affiliations:** Department of Molecular and Integrative Physiology, School of Molecular and Cellular Biology, University of Illinois at Urbana-Champaign, Urbana, IL 61801 USA; Neuroscience Program, University of Illinois at Urbana-Champaign, Urbana, IL 61801 USA

**Keywords:** Mdm2, p53, Nedd4-2, Synchrony, Network activity, Multielectrode array, Seizure

## Abstract

**Background:**

Neural network synchrony is a critical factor in regulating information transmission through the nervous system. Improperly regulated neural network synchrony is implicated in pathophysiological conditions such as epilepsy. Despite the awareness of its importance, the molecular signaling underlying the regulation of neural network synchrony, especially after stimulation, remains largely unknown.

**Results:**

In this study, we show that elevation of neuronal activity by the GABA(A) receptor antagonist, Picrotoxin, increases neural network synchrony in primary mouse cortical neuron cultures. The elevation of neuronal activity triggers Mdm2-dependent degradation of the tumor suppressor p53. We show here that blocking the degradation of p53 further enhances Picrotoxin-induced neural network synchrony, while promoting the inhibition of p53 with a p53 inhibitor reduces Picrotoxin-induced neural network synchrony. These data suggest that Mdm2-p53 signaling mediates a feedback mechanism to fine-tune neural network synchrony after activity stimulation. Furthermore, genetically reducing the expression of a direct target gene of p53, *Nedd4-2*, elevates neural network synchrony basally and occludes the effect of Picrotoxin. Finally, using a kainic acid-induced seizure model in mice, we show that alterations of Mdm2-p53-Nedd4-2 signaling affect seizure susceptibility.

**Conclusion:**

Together, our findings elucidate a critical role of Mdm2-p53-Nedd4-2 signaling underlying the regulation of neural network synchrony and seizure susceptibility and reveal potential therapeutic targets for hyperexcitability-associated neurological disorders.

**Electronic supplementary material:**

The online version of this article (doi:10.1186/s13041-016-0214-6) contains supplementary material, which is available to authorized users.

## Introduction

Neural network activity modulates the efficacy of synaptic transmission, and proper regulation of neural network activity, such as through the modulation of synchronization, has been shown to be important in development [1], learning and memory [2], and disease such as epilepsy [3]. Typical network activity is characterized by sporadic but meaningful spatiotemporal patterns of neuronal firing [4]. However, many seizures are preceded by local high frequency synchronous neuronal firing that can spread throughout the brain [5]. Unfortunately, the molecular changes leading to disrupted neural network synchrony are poorly understood.

One promising pathway involving the feedback, or inhibitory, regulation of network activity after stimulation is Mdm2-p53-Nedd4-2 signaling [6]. When neuronal activity is chronically elevated, phosphorylation of the E3 ligase, Murine Double Minute-2 (Mdm2) leads to the down-regulation of its substrate, p53. The degradation of p53, a transcription factor, leads to the up-regulation of neural precursor cell expressed developmentally down-regulated gene 4-like (*Nedd4-2*) [7]. *Nedd4-2* is an epilepsy-associated gene [8] encoding a ubiquitin E3 ligase with many membrane receptor and ion channel targets [6, 9, 10]. However, it is unclear whether and how Nedd4-2 and its upstream Mdm2-p53 signaling regulate neural network synchrony and, most importantly, seizure susceptibility.

With a multielectrode array (MEA) recording system and a series of pharmacological and mouse genetic approaches, we found that Mdm2-p53-Nedd4-2 signaling contributes to a feedback modulation of neural network synchrony after chronic neuronal activity stimulation. With a kainic acid-induced seizure model in mice, we also showed that seizure susceptibility is reduced when p53 is pharmacologically inhibited. When using a mouse model with a disrupted form of Nedd4-2, *Nedd4-2*^*andi*^, seizure susceptibility is enhanced and no longer responds to the inhibition of p53. This study illustrates the first feedback regulation of neural network synchrony and provides the first evidence linking both the tumor suppressor p53 and epilepsy-associated gene *Nedd4-2* to seizure susceptibility. These findings imply numerous avenues for regulating neural network synchrony and provide novel targets for studying and potentially treating hyperexcitability-associated neurological disorders such as epilepsy.

## Results

### Mdm2-p53 signaling provides feedback modulation for neural network synchrony during chronic elevation of neuronal activity

**Fig. 1:**
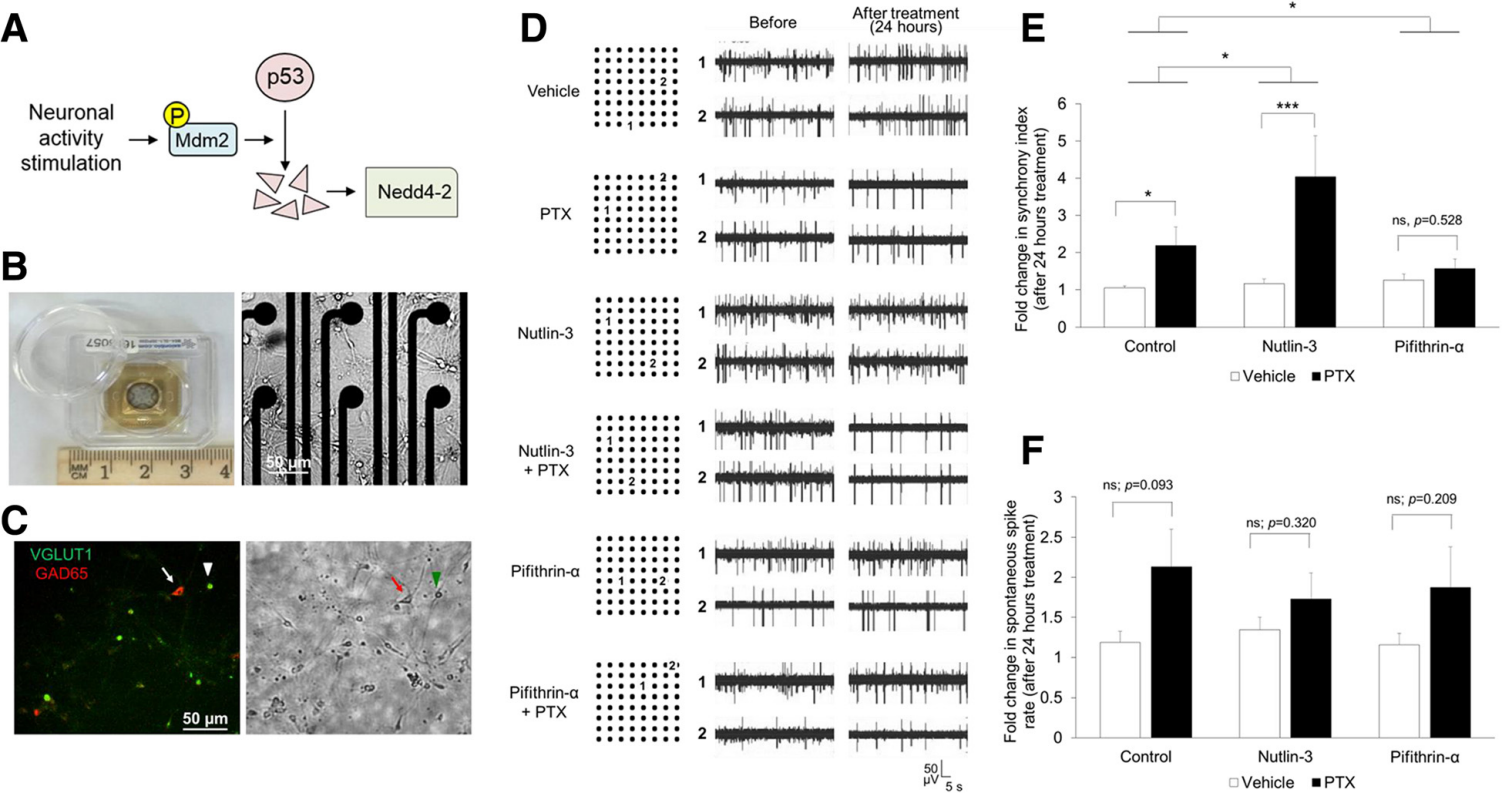
Mdm2-p53 signaling provides feedback modulation for neural network synchrony after stimulation of neuronal activity. **a** A schematic of Mdm2-p53-Nedd4-2 signaling. **b** A picture of a multi-electrode array (MEA) dish (*left*) and a representative image of cultured cortical neurons growing in a MEA dish at DIV 13 (*right*). **c** A representative immunohistochemical image (*left*) and the corresponding bright field picture (*right*) showing a mixture of excitatory neurons (VGLUT1-positive, indicated by a *green arrowhead*) and GABAergic interneurons (GAD65-positive, indicated by a *red arrow*) in our cultures. **d** Representative traces of recordings from 2 electrodes that are over 400 μm away from each other. Selected electrodes are marked with “1” and “2” on the electrode map (*left*). Traces from the same electrodes before or after 24-h treatment are shown on the right. **e** Quantification of synchrony index from entire MEA after 0- or 24-h treatments as indicated. **f** Quantification of spontaneous spike rates (spikes/sec) from entire MEA after 0- or 24-h treatments as indicated. Plotted is the average of “after treatment” normalized to the mean of its own “before treatment” for each MEA. ANOVA with post**-**hoc Tukey test was used. (*n* = 5–8 cultures, *p < 0.05, ***p < 0.001, ns: non-significant)

With the intent of studying the role of Mdm2-p53 signaling in the regulation of neural network synchrony (Fig. 1a), we used an MEA to record extracellular spontaneous spikes (action potentials) of cultured primary mouse cortical neurons (Fig. 1b). This system allowed recording and analysis of spontaneous spikes from the same cultures before and after treatment, greatly diminishing variability between cultures. Cells were obtained from mice at p0 to p1, plated onto MEAs, and recorded at DIV 13 and 14. The population of neurons in our cultures consisted of a mixture of excitatory neurons (VGLUT1 positive; ~95 %) and sparse GABAergic inhibitory neurons (GAD65 positive; ~5 %) (Fig. 1c), as observed in other studies [11, 12]. To determine whether and how neural network synchrony was regulated after stimulation in cultured neurons, we applied a widely used method to elevate neuronal activity; we treated primary mouse (C57BL/6) cortical neuron cultures with the GABA(A) receptor antagonist Picrotoxin (PTX, 100 μM) [13, 14]. When compared with the baseline recording, neural network synchrony is elevated after 24 h of PTX treatment, shown in representative traces from two designated electrodes more than 400 μm away from each other on a single MEA (Fig. 1d) and by quantification of the synchrony index which is based upon the synchrony of spike firing between electrode pairs throughout the entire MEA (Fig. 1e).

We’ve previously shown that chronic elevation of neuronal activity triggers Mdm2-dependent degradation of the tumor suppressor p53 [6]. To determine whether such degradation is involved in the regulation of neural network synchrony, we applied a small-molecule inhibitor, Nutlin-3 (1 μM), that specifically inhibits Mdm2-p53 interaction and Mdm2-mediated p53 degradation [15]. As shown (Fig. 1d-e), Nutlin-3 alone does not affect neural network synchrony. Surprisingly, when cells were treated with Nutlin-3 and PTX, the neural network synchrony is further enhanced in comparison to PTX alone. Because Mdm2-mediated p53 degradation leads to reduction of p53 transcriptional activity [6], to further support the role of Mdm2-p53 signaling in regulating neural network synchrony, we took another approach by applying a specific p53 inhibitor, Pifithrin-α (1 μM) [16], to strengthen the reduction of p53 activity after PTX treatment. As shown (Fig. 1d-e), although Pifithrin-α alone does not affect neural network synchrony, it significantly reduces the elevation of neural network synchrony when applied with PTX. To eliminate potential concern regarding cell death, we determined cell viability and apoptosis and observed no apparent effects in cultures treated with PTX along with Nutlin-3 or Pifithrin-α (Additional file 1: Figure S1).

Our observations in neural network synchrony raised another potential question of whether the synchrony is affected by or correlated with the number of qualifying spikes after treatment. We therefore analyzed the spontaneous spike frequency (average number of spikes/sec). First, we compared the neural network synchrony and spontaneous spike frequency after PTX treatment for 24 and 48 h. Chronic stimulation of neuronal activity leads to multiple homeostatic downscaling mechanisms, such as reduction of spontaneous spike frequency [17], to reduce circuit excitability. As shown (Additional file 2: Table S1), the cultures treated with PTX have significantly lower spike frequency at the 48-h time point when compared to the 24-h time point, indicating a downscaling of firing activity. However, the neural network synchrony is continuously elevated after PTX treatments for 48 h. These data suggest that the elevation of neural network synchrony and the scaling of spike frequency are likely uncoupled events, at least between 24- and 48- h after neuronal activity stimulation. Second, we compared the spontaneous spike frequency in cultures treated with PTX and Nutlin-3 or Pifithrin-α for 24 h. As shown (Fig. 1f), although there is a slight trend toward elevation of spontaneous spike frequency after PTX treatment, neither Nutlin-3, nor Pifithrin-α showed similar effects as in neural network synchrony (Fig. 1e). Although Mdm2 can ubiquitinate another substrate, post-synaptic density protein 95 (PSD-95) [18, 19] and presumably affect spontaneous spike frequency, Nutlin-3 or Pifithrin-α is likely only affecting p53 signaling and thus shows no significant effect on spontaneous spike frequency. Altogether, our data suggest that Mdm2-p53 signaling, which is known to be induced by neuronal activity, provides a feedback modulation of neural network synchrony independent of the changes in spontaneous spike frequency.

### Mdm2-p53 signaling does not affect neural network bursting during chronic elevation of neuronal activity

**Fig. 2:**
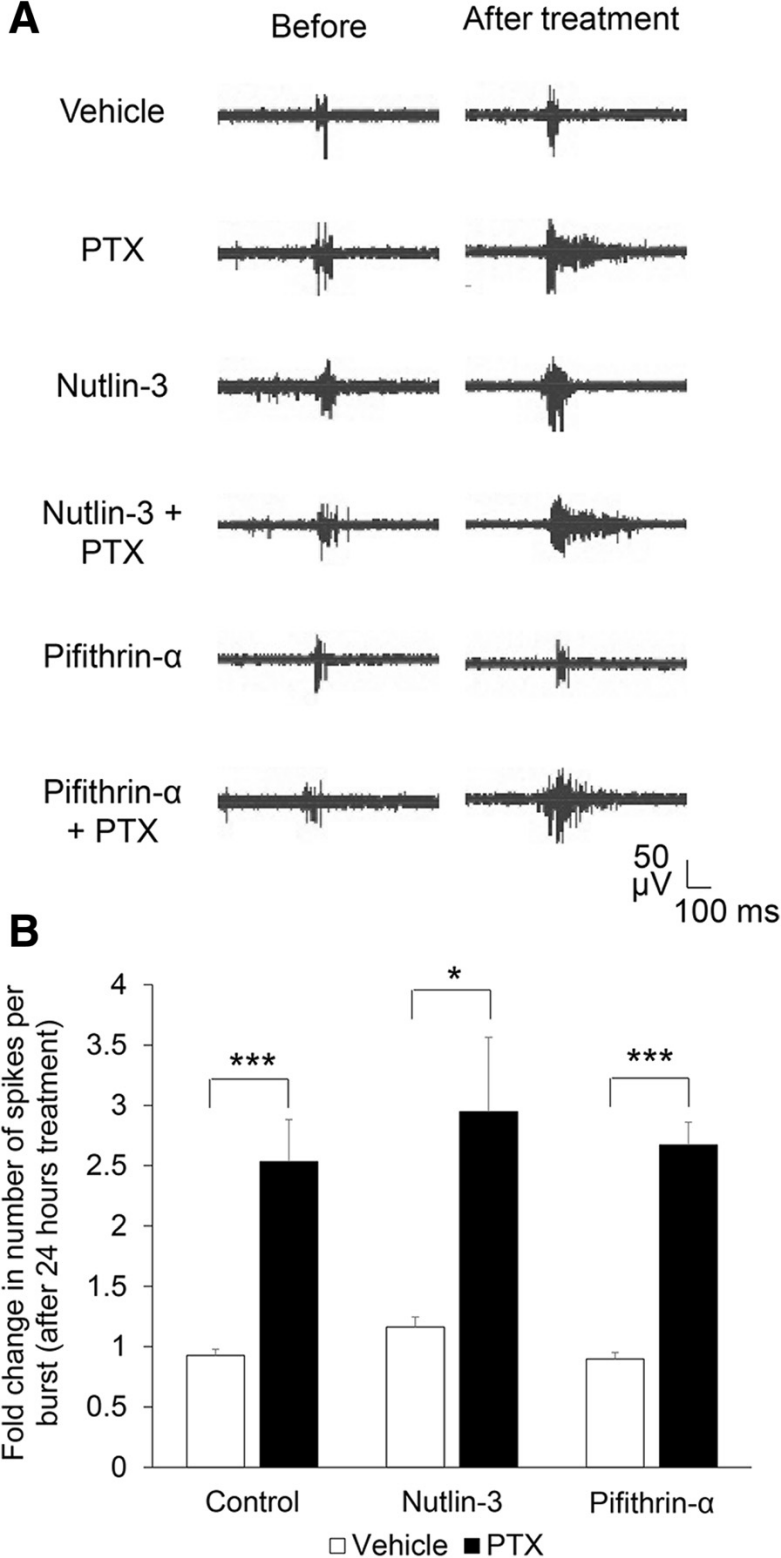
Blocking or promoting Mdm2-p53 signaling does not affect neural network bursting activity before or after neuronal activity stimulation. **a** Representative traces of bursting activity from cultures treated with various agents as indicated. Traces are from a designated electrode before and after treatment. **b** Quantification of the number of spikes per burst after treatment. Plotted is the average of “after treatment” normalized to the mean of its own “before treatment” for each MEA. ANOVA with post**-**hoc Tukey test was used. (*n* = 5–8 cultures, *p < 0.05, ***p < 0.001)

We observed that, after PTX treatment, randomly distributed spikes were clustered (Fig. 1d). This phenomenon represents the induction of bursting activity, which is also known to be elevated by neuronal activity [20]. To evaluate whether Mdm2-p53 signaling also provides a feedback or inhibitory effect on neural network bursting, we analyzed the bursting in our MEA recordings. We defined a burst as a minimum of 5 spikes with a maximum inter-spike interval of 0.1 s. After isolating all the qualifying bursts, we measured the average number of spikes per burst. As shown (Fig. 2a-b), PTX elevated the number of spikes per burst, regardless of whether or not the cells were also treated with Nutlin-3 or Pifithrin-α. These data indicate that Mdm2-p53 signaling does not participate in regulating bursting activity after neuronal activity stimulation and further conclude that Mdm2-p53 signaling selectively mediates neural network synchrony after elevation of neuronal activity.

### Nedd4-2 contributes to the regulation of neural network synchrony

**Fig. 3:**
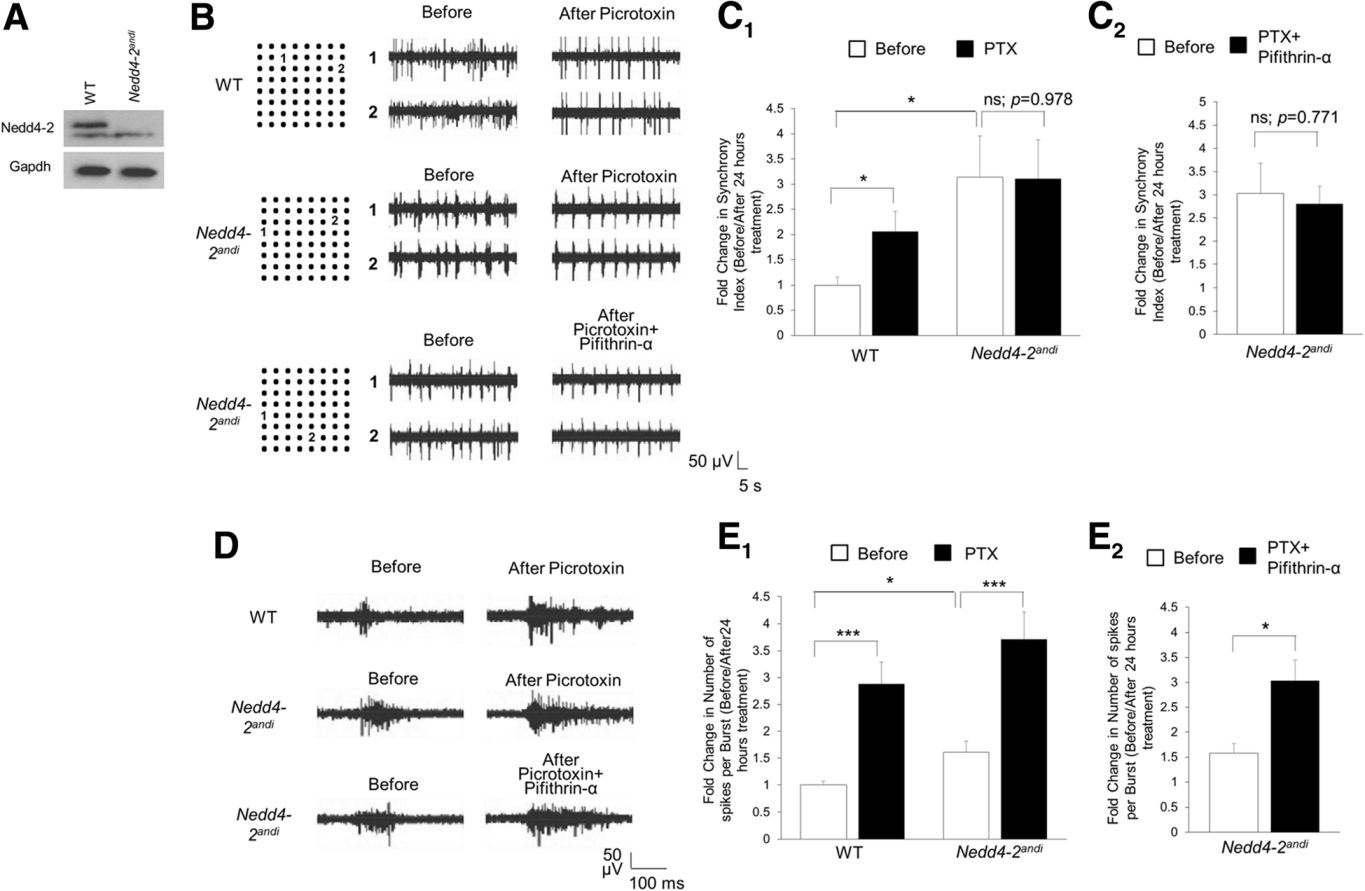
A direct target gene of p53, *Nedd4-2*, mediates neural network synchrony. **a** Representative western blots of Nedd4-2 and Gapdh from cortical lysates of WT or *Nedd4-2* ^*andi*^ mice. **b** Representative traces of recordings from 2 electrodes that are over 400 μm away from each other. Selected electrodes are marked with “1” and “2” on the electrode map (*left*). Traces from the electrodes of WT or *Nedd4-2* ^*andi*^ cultures before or after 24-h treatments are shown on the right. **c**_1_ Quantification of synchrony index from entire MEA of WT or *Nedd4-2* ^*andi*^ cultures before and after PTX treatments. **c**_2_ Quantification of synchrony index from entire MEA of *Nedd4-2* ^*andi*^ cultures before and after Pifithrin-α + PTX treatment. **d** Representative traces of neural network bursts from WT or *Nedd4-2* ^*andi*^ cultures treated as indicated. Traces are from a designated electrode before and after treatment. **e**_1_ Quantification of the number of spikes per burst from WT or *Nedd4-2* ^*andi*^ cultures before and after PTX treatments. **e**_2_ Quantification of the number of spikes per burst from *Nedd4-2* ^*andi*^ cultures before and after Pifithrin-α + PTX treatment. Plotted is the average data normalized to vehicle control from WT cultures before PTX treatment. ANOVA with post**-**hoc Tukey test was used. (*n* = 4–7 cultures, *p < 0.05, ***p < 0.001, ns: non-significant)

*Nedd4-2* is a direct target gene of p53 [7], and its expression is elevated when p53 is inhibited after chronic PTX treatment [6]. To determine whether Nedd4-2 is also involved in reducing or maintaining neural network synchrony, we used *Nedd4-2*^*andi*^ mice, which, with a BALB/cByJ background, carry a spontaneous mutation in exon 2 of the *Nedd4-2* gene. This mutation results in a knockout of long-form Nedd4-2, the predominant form seen in the mouse cortex (Fig. 3a) [6]. Because *Nedd4-2* knockout mice exhibit perinatal lethality [21], *Nedd4-2*^*andi*^ mice serve as the most powerful yet viable system in which to study Nedd4-2. We first observed significantly elevated neural network synchrony basally in *Nedd4-2*^*andi*^ cultures (Fig. 3b-c), which indicates the importance of Nedd4-2 in maintaining or reducing network synchrony. Importantly, while PTX elevates neural network synchrony in WT cultures, it does not possess any effect in *Nedd4-2*^*andi*^ cultures (Fig. 3b-c). Because we previously observed higher neural network synchrony (Fig. 1d), we do not think the failure of further elevation in *Nedd4-2*^*andi*^ cultures is due to a ceiling effect. However, to further support our hypothesis that Nedd4-2 is required for p53-dependent feedback regulation of neural network synchrony, we applied Pifithrin-α with PTX in *Nedd4-2*^*andi*^ cultures. As shown (Fig. 3b-c), Pifithrin-α does not reduce neural network synchrony in *Nedd4-2*^*andi*^ cultures as in WT cultures (Fig. 1b-c), suggesting Nedd4-2 is required for p53-mediated reduction of neural network synchrony after activity stimulation.

Next, to determine whether unresponsiveness to PTX is a general phenomenon in *Nedd4-2*^*andi*^ cultures, we again evaluated neural network bursting. As shown (Fig. 3d-e), we observed a basally elevated number of spikes per burst in *Nedd4-2*^*andi*^ cultures, indicating Nedd4-2 is required for maintaining network bursting activity. However, both WT and *Nedd4-2*^*andi*^ cultures respond similarly to PTX and show a significantly higher number of spikes per burst after PTX treatment. And, similar to WT cultures, Pifithrin-α does not have any effect on network bursting activity in *Nedd4-2*^*andi*^ cultures (Fig. 3d-e). These data indicate that Nedd4-2, although mediating neural network bursting basally, is not involved in the elevation of neural network bursting after neuronal activity stimulation. Altogether, our findings suggest that the Mdm2-p53-Nedd4-2 signaling pathway selectively contributes to the feedback modulation of neural network synchrony after simulation of neuronal activity.

### Mdm2-p53-Nedd4-2 signaling regulates seizure susceptibility in mice

**Fig. 4:**
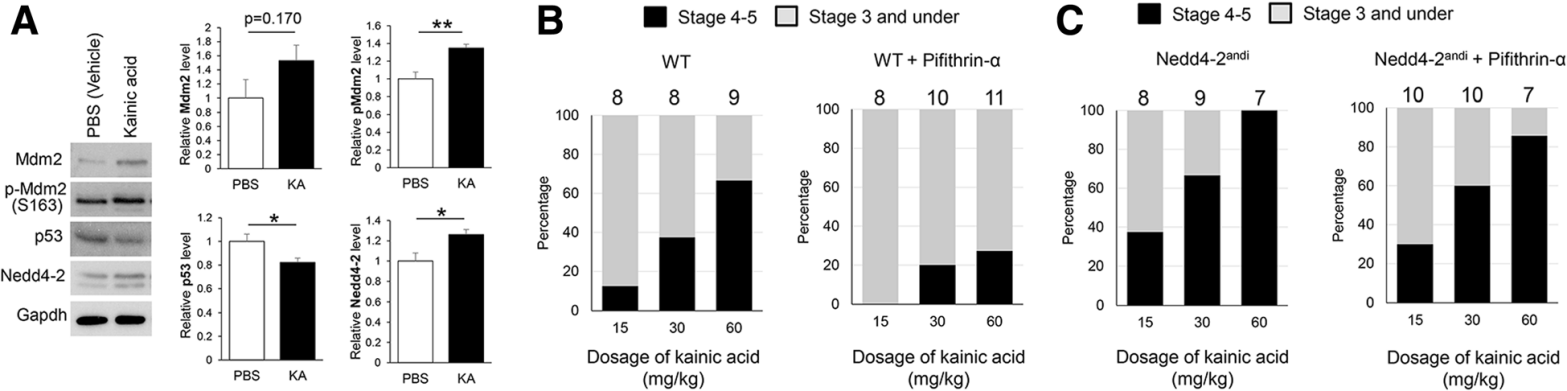
Mdm2-p53-Nedd4-2 signaling regulates seizure susceptibility. **a** Western blots of Mdm2, pMdm2-(S166), p53, Nedd4-2, and Gapdh from cortical lysate of 6–8 weeks old WT C57BL/6 male mice 1 h after kainic acid injection (60 mg/kg). (*n* = 4 littermates per condition, One-sample t-test, *p < 0.05; **p < 0.01). **b** 6–8 weeks old WT (BALB/cByJ background) and **c** *Nedd4-2* ^*andi*^ mice (also in BALB/cByJ background) were intraperitoneally injected with (*right*) or without (*left*) a single dose of Pifithrin-α (2 mg/kg) for 2 days prior to kainic acid injection (15, 30 or 60 mg/kg). The number of mice used in each condition is shown on the top of each bar. Mice showing stage 4 and/or 5 seizure are counted as 1 while mice showing stage 3 seizure or under are counted as 0. The statistical results after ANOVA with post**-**hoc Tukey test are as below: WT vs WT + Pifithrin-α, *p < 0.05; WT vs *Nedd4-2* ^*andi*^, *p < 0.05; *Nedd4-2* ^*andi*^ vs *Nedd4-2* ^*andi*^ + Pifithrin-α, *p* > 0.05

Elevated neural network synchrony is observed in patients and animal models with seizures and epilepsies [22]. We hypothesized that Mdm2-p53-Nedd4-2 signaling was also involved in the regulation of seizure susceptibility in vivo. To test this hypothesis, we first evaluated this signaling in the cortex of WT mice after intraperitoneal injections with kainic acid, a potent agonist for kainate-class ionotropic glutamate receptors, widely used to induce seizures in animal models [23]. Six- to 8-week-old WT C57BL/6 male mice were injected with saline or kainic acid (60 mg/kg, determined using a preliminary test to trigger seizures in C57BL/6 mice). One hour after injection, all 4 mice receiving kainic acid showed at least stage 3 seizure (forelimb clonus) according to Racine’s scoring system [24] and survived. None of the mice receiving saline showed any sign of seizures. The cortical tissues were then collected, and the results showed an elevation in the signaling of interest. That is, phospho-Mdm2 was elevated, p53 was reduced, and Nedd4-2 was elevated (Fig. 4a). Because Mdm2-p53-Nedd4-2 signaling functions to reduce neural network synchrony, we speculated that facilitating or interrupting this signaling would alter seizure susceptibility accordingly. To test this possibility, we used 6- to 8-week-old WT male mice with the BALB/cByJ background for the purpose of matching the *Nedd4-2*^*andi*^ mice. We first determined the dosage of kainic acid to be used in WT BALB/cByJ mice and then set 15, 30, and 60 mg/kg as our standard dosages because they delivered a wide spectrum of seizure responses within 2 h (Fig. 4b, left). To determine whether p53 signaling was capable of affecting seizure susceptibility, we intraperitoneally injected the p53 inhibitor, Pifithrin-α (2 mg/kg) [25], into the WT mice. Pifithrin-α is known to cross the blood–brain-barrier with high efficiency [26]. Two days after injecting Pifithrin-α to allow sufficient blockage of p53 activity, as previously described [27], we injected the mice with a single dose of kainic acid (15, 30, or 60 mg/kg). As shown (Fig. 4b, right), mice receiving Pifithrin-α had reduced seizure response in comparison to control mice (Fig. 4b, left; ANOVA with post**-**hoc Tukey test, *p* = 0.045). To investigate the involvement of Nedd4-2 in seizure susceptibility, we performed the same experiment using *Nedd4-2*^*andi*^ mice. As shown (Fig. 4c, left), *Nedd4-2*^*andi*^ mice had elevated seizure susceptibility after kainic acid administration when compared to WT mice (ANOVA with post**-**hoc Tukey test, *p* = 0.041). Most importantly, Pifithrin-α failed to reduce seizure susceptibility in *Nedd4-2*^*andi*^ mice (ANOVA with post**-**hoc Tukey test, *p* = 0.262), supporting our hypothesis that Nedd4-2 acts downstream of p53 to mediate neural network synchrony and seizure susceptibility. In conclusion, our results indicate Mdm2-p53-Nedd4-2 signaling is crucial for regulating neural network synchrony in vitro and seizure susceptibility in vivo.

## Discussion

In this study, we showed that Mdm2-p53-Nedd4-2 signaling provides feedback modulation for neural network synchrony in cultures and seizure susceptibility in mice. To our knowledge, this is the first signaling pathway reported to regulate neural network synchrony, particularly through a feedback mechanism. This is also the first study to experimentally link Nedd4-2 to seizure/epilepsy in mice. Mdm2-mediated p53 degradation is largely affected by Mdm2 phosphorylation at S163/S183. Many pharmacological agents mediating Mdm2 phosphorylation are developed and are potentially beneficial for controlling neural network synchrony and seizure susceptibility. On the other hand, Nedd4-2 can also be a suitable candidate for controlling network synchrony. Several mechanisms have been proposed to explain increased network synchrony after stimulation of neuronal activity, including elevated synapse number [28], elevated post-synaptic strength [29] and enhanced pre-synaptic release probability [30]. Nedd4-2 is known to ubiquitinate multiple ion channels and membrane receptors, including voltage-gated sodium channels [10], voltage-gated potassium channels [9], and AMPA receptors [6]. Ubiquitination, and presumably degradation, of these proteins by Nedd4-2 likely leads to alteration of network activity. Manipulating the functions of Nedd4-2 is therefore a potential strategy for modulating network activity and seizure intensity. However, at this moment, it remains unclear which substrates or downstream signaling of Nedd4-2 is more relevant in the regulation of neural network synchrony. Several missense mutations of Nedd4-2 have been identified in patients with epilepsy [10]. Understanding the defects of those mutations in regard to substrate specificity and network activity regulation could help reveal the pertinent molecular mechanisms downstream of Nedd4-2.

Dysregulated network activity is also associated with other neurological disorders such as autism spectrum disorders (ASDs). In animal models of the two most studied ASDs, Fragile X Syndrome (FXS) and Tuberos Sclerosis Complex (TSC), elevated network activity and seizure, or seizure-like activity, are observed [31–34]. Dysregulated Mdm2 is known to contribute to the deficit of activity-dependent synapse elimination in the FXS mouse model, *Fmr1* knockout mice [18]. In addition, Mdm2 is involved in mechanistic targeting of rapamycin (mTOR) signaling [35, 36], the pathway that is dysregulated and responsible for the deficits in TSC [37]. We showed here that Mdm2 contributes to network activity regulation, indicating that it might be involved in the dysregulated network activity seen in FXS and TSC. One potential mechanism is through Akt-mediated phosphorylation of Mdm2. We previously showed that PTX-induced phosphorylation of Mdm2 is primarily mediated by Akt [6]. Because Akt activity is altered in both FXS and TSC [38, 39], it could render Mdm2 unresponsive to phosphorylation when the cue for reducing network activity is presented. Recently, studies have proposed targeting PI3K/Akt signaling to correct circuit hyperexcitability and intellectual deficits in FXS [40, 41]. Because Mdm2 is a direct target of PI3K/Akt signaling, our study provides another potential explanation for the hyperexcitability associated with dysregulated PI3K/Akt signaling in ASDs. If this is true, pending future studies, Mdm2-p53 signaling could provide novel therapeutic targets for treating or controlling some symptoms associated with ASDs.

## Materials and methods

### Animals and primary cortical neuron cultures

Wild-type (WT) mice in C57BL/6 background were purchased from Charles River. The *Nedd4-2*^*andi*^ mice and WT control mice in BALB/cByJ background were purchased from The Jackson Laboratory. Primary cortical neuron cultures were made from p0-p1 mice as described previously [18]. All experiments followed the guidelines provided by the Illinois Institutional Animal Care and Use Committee to minimize animal suffering and the number of animals used.

### Reagents

Picrotoxin (PTX) was from Santa Cruz Biotechnology. Dimethyl sulfoxide (DMSO) was from Fisher Scientific. Pifithrin-α was from Adipogen Corporation. Nutlin-3 was from Cayman Chemical. PTX was dissolved in methanol while Pifithrin-α and Nutlin-3 were dissolved in DMSO. DMSO and methanol were therefore used as vehicles in this study. The antibodies used in this study were purchased from Santa Cruz Biotechnology (anti-p53 and anti-Mdm2), Cell Signaling (anti-Nedd4-2 and anti-phospho-Mdm2) GenScript Corporation (anti-Gapdh), StressMarq (anti-VGLUT1) and Abgent (anti-GAD65).

### Cell viability and apoptosis assays

Cell viability, determined by the activity of live-cell proteases, and apoptosis, determined by the activity of caspase-3/7, were measured using ApoTox-Glo™ Triplex Assay (Promega) with enzyme-linked immunosorbent assay (ELISA) as described in the manufacturer’s manual.

### Immunohistochemistry

Immunohistochemistry was performed as described [18]. In brief, dissociated neurons were fixed at DIV 14 with ice-cold buffer (4 % paraformaldehyde and 5 % sucrose in PBS) for 15 min followed by permeabilization with 0.5 % Triton X-100 in PBS. After blocking (1 % BSA in PBS) for 30 min, primary antibodies (anti-VGLUT1 and anti-GAD65) were diluted 1:200 in blocking buffer and applied to the fixed cells overnight at 4 °C. After washing three times with PBS, fluorescence-conjugated secondary antibodies (Invitrogen) were applied to the cells at room temperature for 3 h. After additionally washing the cells three times with PBS, the coverslips were mounted and imaged on a Zeiss LSM 700 confocal microscope.

### Western blotting

After SDS-PAGE, the gel was transferred onto a polyvinylidene fluoride membrane. After blocking with a 1 % Bovine Serum Albumins in TBST buffer (20 mM Tris pH 7.5, 150 mM NaCl, 0.1 % Tween-20), the membrane was incubated with primary antibody overnight at 4 °C, followed by a 30-min washing with PBS. The membrane was then incubated with an HRP-conjugated secondary antibody (from Santa Cruz Biotechnology) for 1 h at room temperature, followed by another 30-min washing. Finally, the membrane was developed with an ECL Chemiluminescent Reagent [6].

### MEA recording

All multi-unit extracellular recordings were done using an Axion Muse 64-channel system in single well MEAs (M64-GL1-30Pt200, Axion Biosystems) inside a 5 % CO_2_, 37 °C recording incubator. Field potentials (voltage) at each electrode relative to the ground electrode were recorded with a sampling rate of 25 kHz. After 30 min of recording, the MEA was returned to the 5 % CO_2_, 37 °C, humidified growing incubator. Due to changes in network activity caused by physical movement of the MEA, only the last 15 min of each recording were used in data analyses. AxIS software (Axion Biosystems) was used for the extraction of spikes (i.e. action potentials) from the raw electrical signal obtained from the Axion Muse system. After filtering, a threshold of ±6 standard deviations was independently set for each channel; activity exceeding this threshold was counted as a spike. The settings for burst detection were a minimum of 5 spikes with a maximum inter-spike interval of 0.1 s. Synchrony index was computed through AxIS software, based on a previously published algorithm [42], by taking the cross-correlation between two spike trains, removing the portions of the cross-correlogram that are contributed by the auto-correlations of each spike train, and reducing the distribution to a single metric. A value of 0 corresponds to no synchrony and a value of 1 corresponds to perfect synchrony. For each experiment a baseline was recorded for 30 min at Days-In-Vitro (DIV) 13. Then Pifithrin-α (1 μM), Nutlin-3 (1 μM) and/or PTX (100 μM) was added. Twenty-four hours after the start of the baseline recording, the cells were recorded again for 30 min. Representative traces were selected from two electrodes with a minimum firing rate of 0.5 Hz and are at least 400 μm away from each other. The analysis and quantification of synchrony index, however, were derived from the entire MEA.

### Seizure susceptibility

Male mice at age 6–8 weeks old were intraperitoneally injected with kainic acid, prepared in saline solution (Hannas Pharmaceutical), at the doses of 15, 30, or 60 mg/kg. The total injection volume was kept close to 0.2 ml. After injection, mice were closely observed in real time for 2 h. The intensity of seizure was assessed by Racine’s scoring system [24]. To clearly determine seizure susceptibility, only stage 4 (rearing) and stage 5 (rearing and falling) were considered positive for seizures, as previously performed [43]. Mice showing stage 4 seizure and above are counted as 1, while mice showing stage 3 seizure or under are counted as 0 for statistical analysis.

### Statistical analysis

ANOVA with post**-**hoc Tukey HSD (Honest Significant Differences) test was used for the comparison between multiple groups. Student’s t-test was used for analyzing network activity within the same treatment group and for western blotting results. Each “n” indicates an independent culture. Differences are considered significant at the level of *p* < 0.05.

## Additional files

Additional file 1: Figure S1: Primary cortical neuron cultures treated with PTX along with or without Nutlin-3 or Pifithrin-α do not exhibit altered cell viability or apoptosis. (DOCX 78 kb) Additional file 2: Table S1: Summary of normalized MEA measurements of spike rate and synchrony index over 48 h of recording after elevation of neuronal activity. (DOCX 15 kb)

## Abbreviation

KA: kainic acid
Mdm2: murine double minute-2
MEA: multielectrode array
Nedd4-2: neural precursor cell expressed developmentally down-regulated gene 4-like
PTX: picrotoxin
WT: wildtype
