# Supplementary material for: Feedback modulation of neural network synchrony and seizure susceptibility by Mdm2-p53-Nedd4-2 signaling
Source: Mol Brain. 2016 Mar 22;9:32. doi: 10.1186/s13041-016-0214-6 (PMC4802718; doi:10.1186/s13041-016-0214-6)

**Additional file 1: Figure S1.**

Primary cortical neuron cultures treated with PTX along with or without Nutlin-3 or Pifithrin-α do not exhibit altered cell viability (A) or apoptosis (B). Culture medium alone serves as the blank (gray bars). (n = 6)


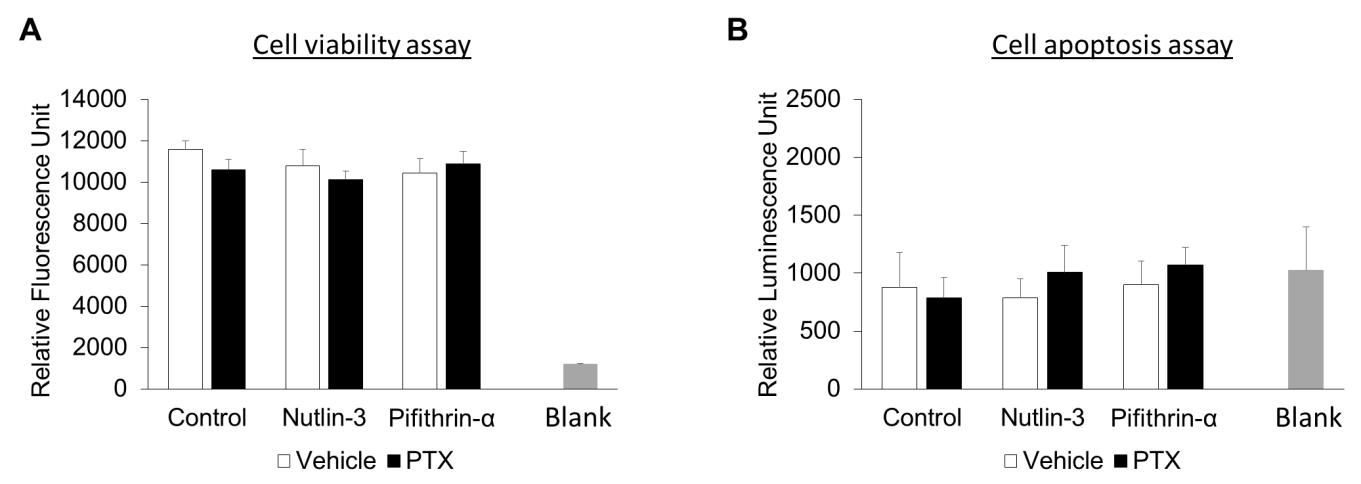

Supplement: Additional file 1: Figure S1: — Primary cortical neuron cultures treated with PTX along with or without Nutlin-3 or Pifithrin-α do not exhibit altered cell viability or apoptosis. (DOCX 78 kb) [file 13041_2016_214_MOESM1_ESM.docx]
