# Supplementary material for: Feedback modulation of neural network synchrony and seizure susceptibility by Mdm2-p53-Nedd4-2 signaling
Source: Mol Brain. 2016 Mar 22;9:32. doi: 10.1186/s13041-016-0214-6 (PMC4802718; doi:10.1186/s13041-016-0214-6)
Supplement: Additional file 2: Table S1: — Summary of normalized MEA measurements of spike rate and synchrony index over 48 h of recording after elevation of neuronal activity. (DOCX 15 kb) [file 13041_2016_214_MOESM2_ESM.docx]

**Additional file 2: Table S1. Summary of normalized MEA measurements of spike rate and synchrony index over 48 hours of recording after elevation of neuronal activity**

**Spontaneous Spike Rate**

| Experiment | 24 hours treatment | 48 hours treatment | Statistical analysis |
| --- | --- | --- | --- |
| Vehicle (MeOH) | 1.184 ± 0.143 (7) | 1.478 ± 0.272 (7) | *p* = 0.494 |
| PTX | 2.133 ± 0.470 (7) | 1.413 ± 0.982 (7) | * *p* = 0.032 |

**Synchrony Index**

| Experiment | 24 hours treatment | 48 hours treatment | Statistical analysis |
| --- | --- | --- | --- |
| Vehicle (MeOH) | 1.019 ± 0.057 (5) | 1.186 ± 0.017 (5) | * *p* = 0.045^†^ |
| PTX | 2.190 ± 0.498 (6) | 2.308 ± 0.995 (6) | *p* = 0.442 |

^†^The slight increase of synchrony index in vehicle treated cultures is likely contributed by the enhanced maturity and density of neuronal processes over time.

Data are presented as mean ± SEM after normalized to the baseline (0-hour); number of MEAs are indicated in parentheses. Paired t-test is used to determine the difference between 24- and 48-hour treatments. The data of 24 hours treatment were presented in Fig. 1E and 1F.
